# Calcium Stimulates Self-Assembly of Protein Kinase C α *In Vitro*

**DOI:** 10.1371/journal.pone.0162331

**Published:** 2016-10-05

**Authors:** Carter J. Swanson, Ruth F. Sommese, Karl J. Petersen, Michael Ritt, Joshua Karslake, David D. Thomas, Sivaraj Sivaramakrishnan

**Affiliations:** 1 Biophysics Program, University of Michigan, Ann Arbor, 48109, United States of America; 2 Dept. of Genetics, Cell Biology and Development, University of Minnesota, Twin Cities, 55455, United States of America; 3 Dept. of Biochemistry, Molecular Biology, and Biophysics, University of Minnesota, Twin Cities, 55455, United States of America; Wayne State University, UNITED STATES

## Abstract

Protein kinase C α (PKCα) is a nodal regulator in several intracellular signaling networks. PKCα is composed of modular domains that interact with each other to dynamically regulate spatial-temporal function. We find that PKCα specifically, rapidly and reversibly self-assembles in the presence of calcium *in vitro*. This phenomenon is dependent on, and can be modulated by an intramolecular interaction between the C1a and C2 protein domains of PKCα. Next, we monitor self-assembly of PKC—mCitrine fusion proteins using time-resolved and steady-state homoFRET. HomoFRET between full-length PKCα molecules is observed when in solution with both calcium and liposomes containing either diacylglycerol (DAG) or phosphatidylinositol 4,5-bisphosphate (PI(4,5)P_2_). Surprisingly, the C2 domain is sufficient to cluster on liposomes containing PI(4,5)P_2_, indicating the C1a domain is not required for self-assembly in this context. We conclude that three distinct clustered states of PKCα can be formed depending on what combination of cofactors are bound, but Ca^2+^ is minimally required and sufficient for clustering.

## Introduction

Signal transduction in cells emerges from transient protein interactions that occur in highly crowded cytoplasmic and plasma membrane environments [1, 2]. Pairing of signaling input and output is often achieved by compartmentalization of molecules on a membrane or protein scaffold [3]. An orthogonal mechanism for segregating molecular components involves the self-assembly of macro-scale complexes nucleated by one or more proteins [4]. While such self-assembled macro-molecular complexes are fairly common in cellular processes such as vesicle trafficking [5], cytoskeletal organization [6], and cell division [7] they are much less appreciated in the context of cell signaling and form the focus of this study.

The PKC family of protein kinases functions as a nodal regulator in signaling networks [8]. PKCs function at multiple locations within the cell and tune their function to couple diverse stimuli to distinct outputs [9]. The diversity of PKC function is achieved, in part, by a series of modular domains linked to the catalytic domain. These modular domains render PKC sensitive to different factors. For instance the C2 domain in PKC binds Ca^2+^ ions, phosphatidylserine (PS) and PI(4,5)P_2_, whereas the C1 domains bind diacylglycerol (DAG), and the catalytic domain binds nucleotides and phosphosubstrates [8].

Several researchers have documented the sub-cellular focal accumulation of fluorescently labeled PKCs in response to distinct stimuli and have suggested this as a mechanism to localize and multiplex PKC function in response to different inputs [10–13]. The molecular mechanisms driving these events and the underlying biomolecules that lead to PKC ‘clustering’ remain unknown. Here, we test the hypothesis that interactions between the modular domains of PKC are necessary and sufficient to drive self-assembly of PKC into clusters without the need for membrane scaffolds or other regulatory co-factors.

In this study, we report that PKCα self-assembles into large clusters *in vitro* in the presence of calcium. Self-assembly of PKCα is reversible, it occurs at calcium concentrations consistent with reported Ca^2+^- C2 domain binding, and is largely abolished by a single point-mutation in the C2 domain. Endogenous PKCα in clarified HEK lysates differentially fractionates in the presence of calcium, consistent with self-assembly. We find that PKCα clustering requires at least the C1a and C2 domains. The number of clusters can be modulated by the strength of the C1a-C2 intramolecular interaction, suggesting a mechanism to regulate this phenomenon. Self-assembly of C1a-C2 occurs on times scales < 1 s both *in vitro* and in cells. Finally, we monitor clustering of PKC on liposomes by measuring homoFRET. In the presence of calcium and liposomes containing either DAG or PI(4,5)P_2_ homoFRET is observed between PKCα fusion proteins. Interestingly, only the C2 domain is required to cluster on liposomes containing PI(4,5)P_2_ indicating that, in this context, the C1a domain is not necessary for self-assembly. We conclude that three distinct clustered states of PKCα can be formed depending on what combination of cofactors are bound, but that Ca^2+^ is minimally required.

## Materials and Methods

### DNA constructs

All full-length constructs used in this study with human PKCα were previously reported and shown to be functional and to be appropriately phosphorylated [14]. The pBiex1 (Novagen) plasmid vector was used for Sf9 expression and pcDNA/FRT (Invitrogen) was used for CHO expression. All constructs contained a C-terminal FLAG tag for purification. (Gly-Ser-Gly)_2_ linkers were inserted between each fusion element for rotational freedom. The C1a-C2 biosensors (with three different lengths of SPASM linkers) were sub-cloned with restriction enzymes into the SPASM backbone as previously described [15]. The C1a domain is defined as aa 32–100, C1b as aa 101–151, and C2 as aa 158–292. Site-directed mutagenesis was performed with Pfu-turbo (Agilent).

### Cell lines

Sf9, CHO Flp-In and HEK293T Flp-In are all from ThermoFisher Scientific.

### Sf9 protein expression and purification

Sf9 protein expression and purification was performed as previously described [14]. Briefly, Sf9 cells in suspension were transiently transfected with desired DNA constructs using Escort IV transfection reagent (Sigma-Aldrich) and harvested 60–72 h later. Protein was batch purified with anti-FLAG M2 Affinity gel (Sigma-Aldrich), eluted with FLAG peptide, and buffer-exchanged using Zeba Spin Desalting columns (Pierce) into the working buffer (20 mM HEPES, 0.5 mM EGTA, 5 mM MgCl_2_, 2 mM dithiothreitol (DTT), pH 7.5). Protein was further diluted into this working buffer plus 0.1 mg/ml BSA for all experiments unless otherwise described. Prior to each experiment, de-salted protein was centrifuged (2.8 x 10^5^ rcf, 10 min, 4°C) and the concentration was quantified using either an absorbance at 280 nm and a extinction coefficient calculated using ProtParam (http://web.expasy.org/ProtParam) or using mCitrine (mCit) fluorescence fit to a standard curve (FluoroMax-4, Horiba Scientific). Protein batches were made fresh and experiments performed within 72 hours of Sf9 cell harvesting.

### Fluorescence coverslip assay

#### Data acquisition

All experiments contained 200 nM of PKCα-mCit incubated at 25°C unless otherwise stated. For calcium cycling experiments, an aliquot of stock sample was incubated at a final free calcium concentration of 300 μM for 10 min. A small amount was removed for imaging, and the rest was incubated with saturating EGTA (final 1 mM) for 10 min. Again a small amount was removed for imaging. For imaging, each sample was gently sandwiched between an ethanol cleaned slide and coverslip and sealed by valap (vasoline/lanolin/paraffin). Slides were imaged on a Nikon TiE equipped with a mercury arc lamp, a yellow GFP filter cube (Nikon; 500/20 nm excitation, 515 nm LP, 535/30 nm emission), perfect focus system (Nikon), a 100X 1.4 NA Plan-Apo oil-immersion objective (Nikon), Evolve EMCCD camera (Photometrics), and the Nikon NIS-elements software. Image planes were focused on the coverslip surface and ND filters and exposure time were adjusted to avoid saturation before being held constant for all slides in the matched experiment. Stage translation was performed with the shutter closed to avoid field of view bias and images were taken at 5–15 locations per slide. The free calcium concentration was assessed using MAXCHELATOR (http://maxchelator.stanford.edu/CaEGTA-TS.html). For experiments where mCerulean (mCer) and mCit were monitored, a dual view module (Photometrics; 505 nm beam splitter, D480/30 nm and 535/40nm) designed to monitor mCer and mCit emission was used, and two images were sequentially taken with mCer excitation (custom filter cube 436/20 nm excitation, 455 nm LP) followed by mCit excitation (see above). Only the mCer excitation/–mCer emission and mCit excitation–mCit emission channels were analyzed. Visual inspection of mCer-mCit and mCit-mCer channels showed negligible bleed-through and cross-excitation.

#### Spot identifier

Coverslip assays were quantified with custom Matlab code. Briefly, a background slide was subtracted and the image was median filtered. Spots were identified based on local intensity ratios (center pixel intensity *I*_*center*_ > 1.12 ⟨*I*⟩_*local*_ where ⟨*I*⟩_*local*_ is the mean intensity of surrounding 21 x 21 pixels; only one pixel / neighborhood). Spot intensities were quantified as the sum of pixels with greater than half maximum difference in intensity:

$$I_{spot}=\sum_{x=-10}^{10}\sum_{y=-10}^{10}I_{xy}^{\prime }$$

$$I_{xy}^{\prime }=\left\{ \begin{array}{c} I_{xy}\;:\;I_{xy}>\frac{I_{center}+{\langle I\rangle }_{local}}{2}\\ 0\;:\;Otherwise \end{array}\right.$$

Differential fractionation: The indicated protein (300 nM) was fractionated with a 30 min spin at 2.8 x 10^5^ rcf at 22°C. The supernatant was separated, and the ‘pellet’ fraction was resuspended by pipetting in an equal volume of matched buffer. An equal volume aliquot was retained from each fraction for subsequent analysis, and the supernatant fraction was brought to a free calcium concentration of 300 μM and incubated at 22°C for 10 min before a second fractionation (30 min, 2.8 x 10^5^ rcf at 22°C). Following fractionation, the supernatant was separated, and the pellet fraction was resuspended in an equal volume of matched buffer without free calcium. Aliquots were retained, and the pellet fraction volume was adjusted for a final EGTA concentration of 700 μM and incubated for 10 min at 22°C. A third fractionation was performed with matched conditions (30 min, 2.8 x 10^5^ rcf at 22°C). All aliquots were separated by SDS-PAGE, and, for the PKCα-mCit-FLAG experiment, visualized by mCit fluorescence (Typhoon imager, GE Life Sciences), and the PKCα-FLAG transferred and probed with a PKCα specific antibody (see next section for Western analysis). Fractionation was quantified in ImageJ by manually selecting regions, subtracting backgrounds, and comparing the band intensities in the pellet and supernatant fractions.

#### HEK lysate experiment

HEK cells were trypsinized and resuspended in DMEM containing 10% FBS (ThermoFisher Scientific) and Glutamax (ThermoFisher Scientific) and pelleted by low speed centrifugation (250 rcf) at 22°C. The cells were resuspended (~ 5 fold dilution) in working buffer containing 5 μg/ml Aprotinin, 5 μg/ml Leupeptin, 50 μg/ml phenylmethylsulfonyl fluoride, 2 mM DTT, pH 7.5, and incubated with rotation at 4°C for 20 min before mechanical lysis with a 26 gauge 1 mL syringe. All subsequent steps were carried out at 4°C. The lysate was spun at 2.8 x 10^5^ rcf for 30 min and the soluble fraction (clarified lysate) was immediately separated from the pellet fraction. The clarified lysate was treated with 800 μM of EGTA or CaCl_2_ incubated for 3 min and fractionated (2.8 x 10^5^ rcf for 30 min). The supernatant was removed and the pelleted fraction was resuspended in a volume of matching buffer equal to the supernatant. Samples were separated by 10% SDS-PAGE before being transferred to PVDF membrane for 3 h at 300 mA. Orthogonally, a standard curve was made to quantify lipid concentration in the supernatant before and after the clarification spin. The standard relied on the increased fluorescence of DiI (ThermoFisher Scientific) in hydrophobic environments. Blots were blocked with 2% BSA/TBS + 0.1% Tween (TBST) for 1 h at room temperature. Primary PKCα antibody (sc-8393, Santa Cruz Biotechnology, 1:10,000) was added and incubated overnight at 4°C. Blots were washed with TBST and incubated for 1 h with secondary (goat anti-Rabbit—Jackson ImmunoResearch Laboratories, Inc., 1:10,000 in 2% BSA/TBST). Blots were washed with TBST, developed with Immobilin Western chemiluminescent HRP substrate (Millipore), and imaged with ChemiDoc-it imaging system (UVP). Silver-stain analysis performed according to manufacturer’s protocol (Pierce).

### Dynamic light scattering

DLS data was collected on a DynaPro NanoStar (Wyatt Technology) at 25°C. Protein (1 μM) was in the working buffer with no BSA added. Sequentially, the buffer was brought to a final concentration of 800 μM CaCl_2_ and 1 mM EGTA using concentrated stocks at (less than 2.5% of the final volume of buffer was added). Each autocorrelation is the mean of 10 repetitive readings of 10s each, and error bars represent the mean and standard deviation of 3 independent readings. Autocorrelation data were least squares fit to a single exponential decay function (GraphPad) to obtain τ values. Additionally, the regularization and cumulant fits were performed using the Wyatt Technology software platform (Dynamics V7.1.7) utilizing the isotropic spheres model. The mean of the monomer peak was obtained by averaging the MW from each acquisition from 3 batches of protein. The particle mass was determined by a single cumulant fit, and each reading was normalized by the mean of the initial EGTA buffered condition.

### Size-exclusion chromatography

A Superdex 200 10/300 GL Size-exclusion column (GE) on an FPLC (BioRad) was pre-equilibrated with 3 column volumes of HEPES buffer, 100 mM NaCl_2_, 500 μM EGTA (EGTA) or + 800 μM CaCl_2_ (calcium condition). PKCα-mCit or PKCα(D246N)-mCit (9.5 μM) was incubated with matched buffer condition for indicated time and 100 μL was injected and flowed through the column. Fractions were collected (200 μL) in a black clear bottom 96 well plate (Grenier) and scanned for mCit or mCer fluorescence (SpectraMax M5e). Chromatography and pre-incubation of protein were all performed at 4°C. The column was pre-calibrated using known protein standards (Bio-Rad) to analytically determine protein size. Select fractions were run on SDS-PAGE gel and scanned for mCit fluorescence (Typhoon imager), and either probed with PKCα antibody or stained with Coomassie brilliant blue to verify that fractions corresponded with anticipated molecular weights.

### Time-resolved fluorescence anisotropy

We performed and analyzed time-resolved fluorescence anisotropy of mCit data using time-correlated single photon counting and direct waveform recording methods as described previously [16, 17]. A 480 nm laser line with 515 nm LP was used in TCSPC experiments and a 532 nm laser line with 570 nm LP was used for DWR experiments. All time-resolved experiments started with 500 nM of protein, and sequential, polarized measurements of 0°, 54.7° and 90° were recorded for each condition. Using analysis software described previously [18], the single exponential fluorescence lifetime (τ) was first assessed using fluorescent recordings obtained at 54.7°. Subsequently the fluorescence data at 0° and 90° were fit to a 2 exponential decay function using a fixed τ value. Steady-state anisotropies were calculated as the weighted average of the anisotropy decay given by the best-fit model parameters:

$$r=\frac{\int_{0}^{\infty }r(t)F(t)dt}{\int_{0}^{\infty }F(t)dt}=r_{0}\left(\chi_{1}\frac{\phi_{1}}{\phi_{1}+\tau }+(1-\chi_{1})\frac{\phi_{2}}{\phi_{2}+\tau }\right)$$

where *r*_0_ is the initial anisotropy, *ϕ*_1_,*ϕ*_2_ are the correlation times, *χ*_1_ is the fractional contribution to the anisotropy decay, *τ* is the fluorescence lifetime, and the final anisotropy is assumed to be zero.

### Liposome preparation

Liposomes were prepared fresh from chloroform stocks (Avanti) and mol % was calculated using manufacturer provided concentrations and molecular weights. The chloroform was dried with nitrogen and incubation under vacuum for 1 h. The lipids were brought to a working concentration of 1.27 mM with addition of working buffer and were allowed to hydrate at 60°C for 1 h with intermittent suspension by pipetting. Liposomes were generated by 10 rounds of sonication (10 s on, 50 s off). Data presented are representative of multiple batches of liposome preparations, but were all performed with the same stocks of lipid. For the time-resolved anisotropy measurements instead of sonication, the liposome mixture was extruded using a 100 nm nitrocellulose membrane (Millipore) according to manufacturer’s protocol (Avanti).

### Kinase activity

Kinase activity was assessed as previously described [14]. ATP consumption was measured using the Kinase-Glo Max Luminescence assay kit (Promega) measured in a white, opaque 96 well plate (Nunc) on a SpectraMax M5e spectraphotometer (Molecular Devices). Reactions occurred in individual wells with starting concentrations of 100 μM ATP, 100 μM myelin basic protein peptide (4–14; Genscript), 100 nM PKCα-mCit-FLAG, 300 μM free Ca^2+^, and 30 μM of the indicated liposomes. Reactions were initiated with the addition of ATP, and were arrested with addition of the Kinase-Glo substrate after 4 min at room temperature (22.0 ± 0.5°C).

### Steady-state fluorescence anisotropy plate reader

A SpectraMax M5e (Molecular Devices) was used to record fluorescence anisotropy. The instrument was calibrated with FITC (1 μM pH 8.0; excitation 483 nm, emission 515 nm) in solutions of water and 0, 10, 30, 50, 70 and 90% glycerol in a black, clear bottom 96 well plate (n = 12; Greiner). The high and low ends were in good agreement with anticipated anisotropy values (0.046 ± 0.025 and 0.392 ± 0.020) and no correction factor was used (G-factor = 1). These same standards were applied to the microscope where a G-factor was applied (see below). For protein experiments mCit (excitation 485 nm, emission 515 nm) was monitored, and buffer blanks were subtracted before anisotropy calculation. The starting concentration of mCit-containing protein is 200 nM in a 50 μL volume unless otherwise stated, and, for sequential addition of calcium and EGTA, was performed by addition of concentrated stocks (less than 2.5% of the final volume was added). For each condition, 4–8 wells were monitored in triplicate. All experiments were performed at room temperature (22.0 ± 0.5°C).

### Steady-state anisotropy optical set-up

Experiments were collected using a TiE microscope (see above for description). A 40X DIC M/N2 0.75 NA objective combined with the 1.5X optovar (Nikon) was used for imaging cells. FITC calibration samples were placed on glass bottom tissue culture plates (MatTek Corp.) and imaged with polarized excitation and a dual view polarized beam splitter (Photometrics). As with the plate reader, the instrument was calibrated with FITC (1 μM pH 8.0; excitation 483 nm, emission 515 nm) in solutions of water and 0, 10, 30, 50, 70 and 90% glycerol. Qualitatively, the relative anisotropy values matched those obtained from the fluorimeter, but with systematically lower absolute values. A G-factor was applied such that the 0% glycerol sample had an anisotropy value of 0.00 (i.e., equal emission in both polarized channels)[19]. This G-factor (0.773) was applied to all additional anisotropy analysis and no further correction factor was used.

### Mammalian cell imaging

CHO Flp-in cells were cultured as previously described [14]. Cells were transiently transfected with indicated constructs using X-tremeGENE HP (Roche). Cells were transferred to fibronectin (Sigma-Aldrich) coated (1:100 dilution in PBS incubated for 1 hr) glass bottom 35 mm tissue culture plates (MatTek Corp.) 24–48 h post transfection as previously described. Cells were allowed to adhere for 1–4 h in culturing media before cells were imaged. Cells were washed and imaged in freshly prepared HBS (20 mM HEPES, 5mM KCl, 45mM NaCl, 2mM CaCl_2_, 1mM MgCl_2_, 0.2% dextrose, brought to pH 7.4 by NaOH) media. The plates were transferred to the scope and a field of view was manually selected, guided by fluorescence expression and morphology of cells. Cells were imaged every 200 ms or 500 ms over a 5 min time course. Either ionomycin (final 10 μM) or EGTA (final 4 mM) in matched buffer was manually pipetted onto the culture plate during imaging at predetermined time points. Experiments were performed at 22°C. Anisotropy image data was analyzed with custom MatLab code. Both polarized images per frame were registered using the imregister MatLab function. A background region is manually selected, and the mean of the background in each polarization channel is subtracted from the corresponding image. The anisotropy value *r* is calculated using the parallel and perpendicular pixel intensities corrected for the G factor:

$$r=\frac{I_{∥}-G\cdot I_{⊥}}{I_{∥}+2G\cdot I_{⊥}}$$

Anisotropy values above and below theoretical limits are excluded and a mean of all anisotropy values are calculated for each image in the series. For representative images the images were cropped, median filtered and the ‘Fire’ look up table was applied (ImageJ). The correlation coefficient was calculated in the same custom MatLab code as the anisotropy analysis. Following registration and background subtraction, an image of the total mCit intensity (*I*_∥_ + 2*G* · *I*_⊥_) was generated. The corrcoef function was applied between each frame and the first frame. Translocation in this metric is defined by a redistribution of fluorescence intensity within the cell.

### Statistics

All statistics were performed in GraphPad Prism 6. Unless otherwise stated, a student’s unpaired, two-tailed t-test was performed and is represented as symbols corresponding to p-values. n.s. > 0.05; * = 0.05–0.001; ** = 0.001–0.0005; *** = 0.0005–0.0001; **** < 0.0001.

## Results and Discussion

### Calcium induced self-assembly *in vitro*

#### Self-assembly is specific and reversible

**Fig 1:**
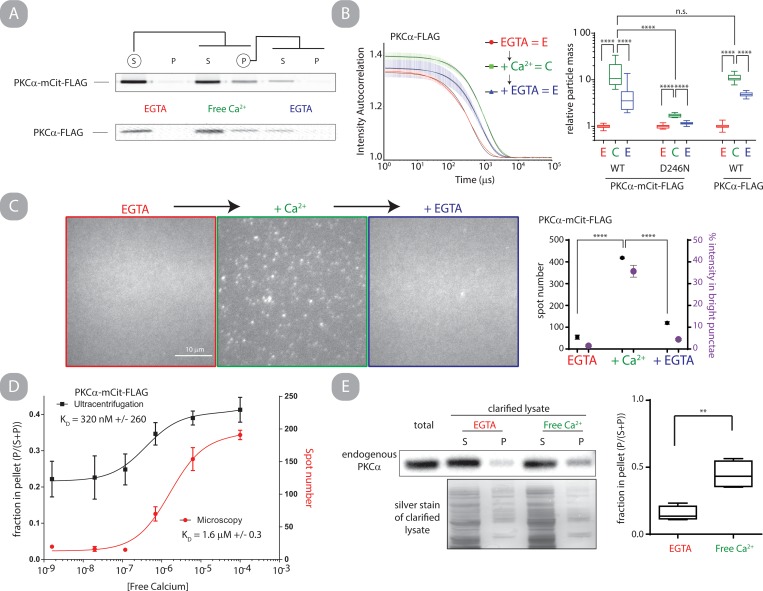
Calcium induces reversible self-assembly of PKCα *in vitro*. A.) Recombinant PKCα-mCit-FLAG or PKCα-FLAG (300 nM) was differentially fractionated into soluble (S) and pellet (P) fractions following high speed centrifugation in two independently performed experiments. Fractionation occurred sequentially in EGTA buffered, free calcium (300 μM), and EGTA buffered solutions where the fractions circled were retained for the subsequent fractionation. Fractions were separated on SDS-PAGE and probed with mCit fluorescence (top) or an anti-PKCα antibody (bottom) in independent experiments. B.) Intensity autocorrelation of dynamic light scattering (DLS) of recombinant PKCα-FLAG (1 μM) sequentially diluted into buffers containing excess EGTA, free calcium, and EGTA with 15 min incubation between readings. The black line is a single exponential fit, and error bars are s.e.m. of 3 independent readings (left). Quantification of the ensemble particle mass normalized to the initial condition of indicated protein from DLS (Right; N ≥ 8, box-and-whisker represents min, max, 25 and 75 percentile and median). C.) Representative fluorescent images of recombinant PKCα-mCit-FLAG (200 nM) sequentially in indicated buffer non-specifically adhered to a glass coverslip. Samples were incubated in buffers for 10 minutes at 22°C before being adhered to slides. (left) Bright spots were identified when the ratio of mCit intensity deviates by >1.12 from the neighboring pixels. Data was quantified from 6 fields of view for each condition (right). D.) Differential sedimentation and spot number on coverslips were assessed as a function of free calcium concentration. The data are least squares fit to a single binding function (solid lines). Error bars represent standard deviation (N = 3 differential sedimentation and N = 5 microscopy). E.) Representative blot and quantification of differential fractionation of endogenous PKCα in 5x diluted and clarified HEK cell lysate (detergent free) probed with anti-PKCα antibody and corresponding silver stain (left) and quantified (Right; N = 4; min, max, 25 and 75 percentile and median).

Previous work from Huang et al suggested that both calcium and phosphatidylserine were necessary for PKCα self-assembly [20]. In contrast, our previous study observed that calcium alone was sufficient for PKCα self-assembly, albeit at an attenuated level [14]. Both of these studies used chemical cross-linking to assess self-assembly, which may be dependent on the conformational state of the protein and bias the conclusions. We chose to re-address whether PKCα self-assembles in the presence of calcium using techniques based on the size and diffusivity of particles including differential sedimentation (**Fig 1A**), dynamic light scattering (DLS) (**Fig 1B**), single-particle fluorescence microscopy (**Fig 1C**) and size-exclusion chromatography (SEC) (**S1 Fig**). Where possible, PKCα self-assembly was assessed without a fluorophore (**Fig 1B and 1C**), and in all cases constructs with fluorophores utilized the monomeric versions of Cerulean and Citrine (mCer and mCit respectively). Overall, each methodology supports our conclusion that PKCα specifically and reversibly oligomerizes in the presence of free calcium *in vitro*. When Ca^2+^ ion coordination is blocked by introducing the D246N point mutation in the C2 domain [21], this self-assembly is significantly reduced compared to wild type (p<0.0001, **Fig 1B and** S1 Fig). Additionally, cycling PKCα between low (< 1 nM) and high (>100 μM) free calcium conditions demonstrates that the phenomenon is partially reversible. Interestingly, the oligomers have several unexpected characteristics. Notably, they are much larger than the previously reported homo-dimers [14, 20]. Following a 30 min incubation in high free calcium conditions, SEC analysis indicates that oligomers are at least 10-fold larger than monomers (**S1 Text**), and DLS analysis supports a mean increase in particle size of ~13-fold (Fig 1B, **S1 Text, S2 Fig**). Second, the number of oligomers, their size, and the fraction of PKCα molecules oligomerized are time- and concentration- dependent. This result is most evident in quantitative analysis of single-particle fluorescence microscopy (**S1 Text, S3 Fig**). We tested if oligomerization is observable when using physiologically relevant free calcium concentrations (<10 μM)[22]. PKCα oligomerization was monitored as a function of free calcium concentration by differential sedimentation and single-particle fluorescence microscopy. In both cases, oligomerization fit well to single-phase binding curves with *K*_*D*_ values falling within physiologically relevant ranges (*K*_*D*_ = 1.6 ± 0.3 μM by microscopy, *K*_*D*_ = 0.32 ± 0.26 μM by sedimentation) (**Fig 1D**).

#### Endogenous mammalian PKCα self-assembles

All of the above experiments used recombinant human PKCα minimally fused with a C-terminal FLAG peptide purification tag. To assess whether the observed self-assembly was an artifact of either Sf9 expression or the FLAG tag, endogenous mammalian expressed PKCα was assessed for differential sedimentation. Pre -clarified HEK cell lysate was fractionated with either free calcium or EGTA and PKCα was probed with a PKCα specific monoclonal antibody **(Fig 1E)**. To assess if the clarifying spin removed the majority of lipid components in the lysate, DiI, a lipophilic small molecule with enhanced fluorescence when intercalated in lipid bilayers, was used to quantify the lipid concentration before and after the spin **(S4 Fig)**. The clarifying spin reduced the lipid concentration by 13.6 fold. A significantly larger fraction of endogenous PKCα pelleted in the presence of free calcium (p < 0.01, **Fig 1E**). This experiment provides direct experimental evidence of altered PKC particle mass in lipid-depleted endogenous cell lysate.

### C1a and C2 domains are minimally sufficient for calcium induced self-assembly

#### The C2 domain is not sufficient for self-assembly

**Fig 2:**
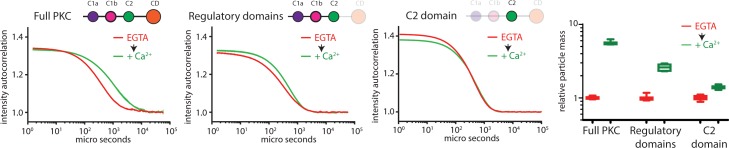
Regulatory domains self-assemble. Mean DLS autocorrelation data before (red) or after addition of free calcium (green) of full-length PKCα (left), the regulatory domains (middle), and C2 domain (right) at matched time points (2 min incubation) and concentration. Normalized particle mass is quantified at the far right. Box and whisker plots represent min, max, 25 and 75 percentile and median of N > 5. Each particle mass increased significantly following calcium addition (p>0.0001).

There have been several suggestions in the literature that C2 domains are capable of oligomerization in a calcium dependent or independent manner [23–28]. To determine the minimal domains required for oligomerization, we created a series of PKCα sensors containing an N-terminal mCer and a C-terminal mCit. In each sensor, a TEV protease site is located between either the V1 and C1a domains, the C1b and C2 domains, or the C2 and the kinase domains. Following TEV proteolysis, we could then identify whether PKCα sensors tagged with the N-terminal, C-terminal, both, or neither fluorescent protein oligomerize in the presence of high levels of free calcium. Using this information, we will be able to identify which polypeptide regions are needed to form fluorescent punctae. Neither the V1 nor the kinase domain was necessary or sufficient for punctae formation. However, separation of the C1 and C2 domains reduced punctae formation in both peptides **(S5 Fig)**. We next assessed the fusion protein with the TEV protease site between the C2 and kinase domains by DLS along with a C2-mCit fusion protein. We pre-treated the construct with TEV protease or BSA, and then measured the relative increase in particle size following a 2 min incubation with free calcium. The three conditions correspond to the panels represented as full-length PKCα, regulatory domains alone, or the C2 domain alone **(Fig 2)**. The intact PKCα sensor has a mean particle size increase of 5.61 ± 0.31 compared to 2.61 ± 0.25 for the regulatory domains alone and 1.40 ± 0.06 for the C2 domain alone. The increase in particle mass of the C2 domain alone is statistically significant, but very modest. Collectively, these data suggest that both the C1 and C2 domains are required to be in the same peptide for self-assembly.

#### C1a and C2 domains self-assemble

**Fig 3:**
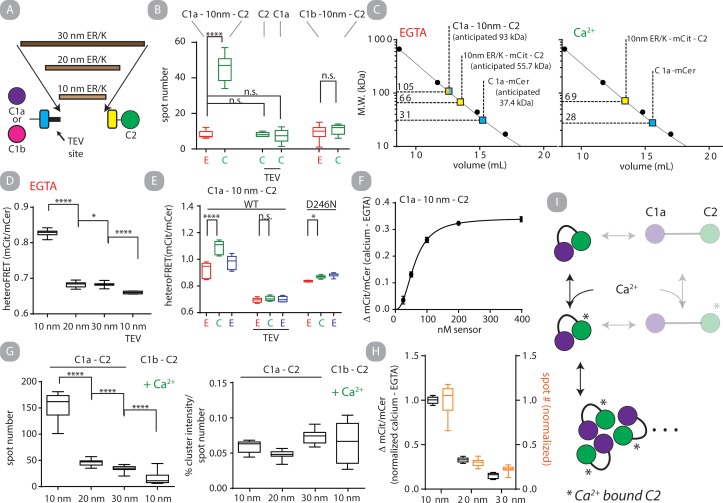
A C1a-C2 complex is minimally sufficient for self-assembly. A.) Schematic for the C1-C2 SPASM biosensors. B.) The C1a and C2 but not C1b and C2 biosensors forms punctae on glass coverslips in the presence of free calcium. The response is abrogated with pretreatment of the biosensor with TEV protease, where neither the N-termini nor C-termini of the biosensor forms punctae. Data from 6 fields of view per condition. C.) Analytical size exclusion chromatography of C1a - 10 nm—C2 in an EGTA buffered (left) or 300 μM free calcium (right) mobile phase. In both conditions the protein was run with or without a pre-incubation with TEV protease. Fluorescence of mCit and mCer in the eluant were assessed. Elution volume of the indicated peptides are compared against molecular standards (black dots) fit to a single exponential decay function (black line). The non-proteolyzed peptide did not come off the column in the presence of calcium. D.) HeteroFRET displayed as the ratio of mCit fluorescence intensity of mCer fluorescence intensity of the indicated biosensors, each at 50 nM in a solution buffered by EGTA. E.) HeteroFRET of C1a - 10 nm—C2 (WT intact or TEV proteolyzed) or D246N (100 nM) sequentially treated free calcium and EGTA. F.) The change in heteroFRET following addition of free calcium for C1a - 10 nm—C2 for the indicated concentration of biosensor. The data are fit to Hill binding model with a hill coefficient of 2.39 ± 0.09 (best fit and standard error; black line). G.) C1a-C2 biosensors with 3 lengths of ER/K linkers are assessed for punctae formation on glass coverslips all in the presence of 300 μM free calcium, data from ≥ 10 fields of view per condition. The longer length linkers systematically reduced punctae formation (middle) with no systematic effect on the relative intensity of punctae (right). H.) Normalized Δ heteroFRET and spot number for C1a –C2 with the indicated ER/K linker length, all fixed at 100 nM. I.) Model demonstrating an intramolecular complex between C1a and C2 is required and sufficient for self-assembly in the presence of free calcium.

It has been reported in the literature that the C2 domain interacts with one or both of the C1 domains [29, 30]. We built two sensors containing the C2 domain and either the C1a or C1b domains separated by either 10 nm, 20 nm or 30 nm SPASM cassettes **(Fig 3A)** [15]. By fluorescence microscopy, we found that only the sensor containing the both C1a and C2 domains formed punctae in the presence of free calcium. Further, when the C1a - 10 nm—C2 protein was cleaved by TEV protease, neither the C1a domain nor the C2 domain peptide formed punctae **(Fig 3B)**. From these results, we hypothesize that an *inter*molecular interaction between C1a and C2 domain is required for self-assembly. Alternatively, the C2 domain and the C1a domain may form homo-dimers or similarly small oligomers that would not be readily detected as punctae. These small homo-dimer oligomers might facilitate cooperative oligomerization in the presence of calcium. We used analytical size chromatography to determine the stoichiometry of both C1a and C2 in the presence and absence of free calcium **(Fig 3C)**. After TEV proteolysis of the C1a - 10 nm—C2 sensor, two peptides are generated, one containing the C1a domain fused to mCer, and the other containing the ER/K helix, mCit and the C2 domain. Fluorescence was used to distinguish the elution profiles of the mCer and mCit containing peptides. The empirically determined mass of both peptides was consistent with the predicted mass of monomers in both conditions. In contrast, non-proteolyzed C1a - 10 nm—C2 in the presence of calcium was not eluted, presumably due to the large size as observed with full-length PKC (S1 Fig). This result is consistent with the hypothesis that an intramolecular interaction between the two domains is required for self-assembly.

#### Basal intramolecular interaction between C1a and C2

It is expected that the C1a and C2 domains interact in the absence of calcium [29]. To test this hypothesis, we compared three sensors containing SPASM cassettes fused between the C1a and C2 domains. The SPASM cassettes have fully extended α-helices with lengths of 10, 20 and 30 nm and are designed, through spontaneous helix breaking, to have *intra*-molecular effective concentrations of 4 μM, 0.4 μM and 0.1 μM respectively [15]. The heteroFRET from these sensors are designed to report on the equilibrium between bound and unbound intramolecular interactions if the total concentration of sensor is << the bi-molecular *K*_*D*_ of the interacting peptides [15]. The heteroFRET is reduced as a function of effective concentration (all constructs at 50 nM) consistent with the hypothesis of an intramolecular C1a-C2 interaction basally **(Fig 3D)**. We tested two putative residues at the C1a-C2 binding interface but point mutations at these residues in the SPASM sensor did not disrupt the interaction (**S6 Fig**) [29].

#### HeteroFRET detects calcium stimulated self-assembly of C1a - 10 nm—C2

We considered if heteroFRET of C1a - 10 nm—C2 sensor could additionally report on calcium stimulated self-assembly. We observed that heteroFRET of C1a - 10 nm—C2 increased and decreased after sequential treatment with free calcium and EGTA **(Fig 3E)**. In contrast, no increase in heteroFRET was observed if C1a - 10 nm—C2 was TEV proteolyzed, and only a modest but significant increase was observed if the D246N point mutation was inserted into the sensor. To distinguish the *intra*molecular and *inter*molecular contributions to heteroFRET, the concentration dependency was assessed. The increase in heteroFRET after addition of free calcium is plotted as a function of sensor concentration (**Fig 3F**). This data is fit well by a Hill binding model, providing a half-maximal value of 61.1 ± 1.2 nM (best fit and standard error), which serves as an estimate of the free in solution equilibrium dissociation constant. These results demonstrate that two factors, intramolecular and intermolecular interactions contribute to observed heteroFRET in the presence of calcium.

#### Intramolecular C1a –C2 interaction regulates self-assembly

We next wanted to explore the dependency of self-assembly on the intramolecular interaction between C1a and C2. Using the fluorescence microscopy assay we found that the number, but not the size of self-assembled punctae was dependent on the effective concentration of C1a –C2 as dictated by the length of ER/K helix **(Fig 3G)**. We additionally monitored the increase in heteroFRET upon free calcium addition for the same constructs. We found that both metrics of self-assembly were highly correlated **(Fig 3H)** (linear correlation r^2^ = 0.936). From these data we propose a model in which (*i*) the C1a and C2 domains are the minimal unit required for self-assembly and(*ii*) an intra-molecular C1a·C2 interaction is required for self-assembly **(Fig 3I)**.

### Monitoring PKCα self-assembly by homoFRET

As heteroFRET is sensitive to both *intra*- and *inter*-molecular interactions in our sensors, we used homoFRET of mCit to specifically report on inter-molecular FRET. HomoFRET in fluorescent proteins is assessed by the fast (τ < 1 ns) depolarization of fluorescence, whereas in the absence of FRET only depolarization on the time scale of rotational diffusion will be observed (τ ~ 15 ns)[31, 32]. Using time-correlated single photon counting (TCSPC) and direct waveform recording (DWR) [16] we can monitor anisotropy up to 15 ns after an excitation pulse is applied to a sample. This time-resolved anisotropy can be used to differentiate between the two modes of depolarization and detect contributions from homoFRET.

**Fig 4:**
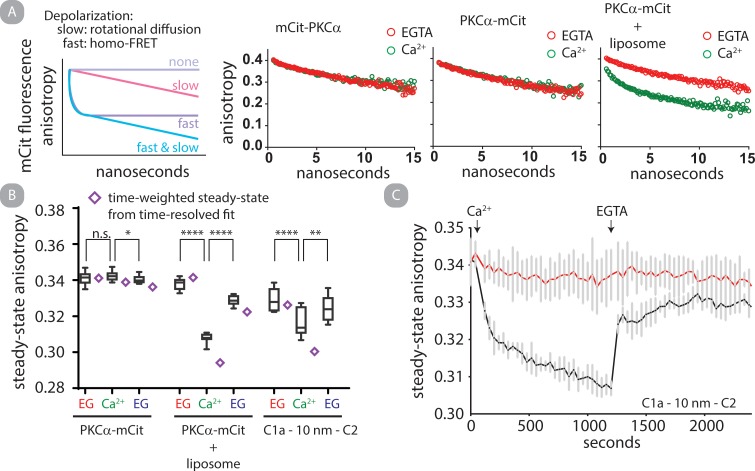
HomoFRET detects PKCα clustering on liposomes but not in solution. Fluorescence anisotropy can be used to monitor homoFRET. A characteristic feature of homoFRET in fluorescent proteins is a depolarization of fluorescence on the time scale of energy migration (10^−10^–10^−9^ seconds) in addition to depolarization occurring from rotational diffusion (tau > 10^−8^ seconds). The two effects on depolarization can be distinguished with time resolved anisotropy measurements (TCSPC or DWR). A.) Representative time-correlated single photon counting (TCSPC) data of mCit—PKCα-FLAG, PKCα-mCit-FLAG and PKCα-mCit-FLAG plus liposomes with and without free calcium. B.) Steady-state fluorescence anisotropy of mCit in C1a-SPASM-C2, PKCα-mCit and PKCα-mCit + liposomes as the protein is sequentially treated with excess EGTA, free calcium and EGTA (N = 16 for steady state measurements). The purple diamonds are simulated steady-state anisotropy values derived from a two-exponential fit of direct waveform recording (DWR) time-resolved anisotropy measurements (see methods). The unilaminar liposome contained 88%PC:10%PS:2%DAG (molar %; PS to PKC molar ratio 32:1) for A and B. C.) Time course of mCit steady-state fluorescence anisotropy from C1a –SPASM—C2 following injection of free calcium (40 s) and EGTA (1200 s) (black line) or buffer blanks (red line). Shown in mean and standard deviation, N = 4 independent matched time course.

We assessed PKCα-mCit, PKCα-mCit + liposomes, mCit-PKCα, and C1a -10 nm SPASM- C2 with and without free calcium. In the PKCα-mCit + liposomes as well as the C1a-10 nm SPASM-C2 conditions, the addition of free calcium clearly results in a fast depolarization consistent with homoFRET **(Fig 4A and 4B; S1 Table**). However, we do not observe homoFRET for PKCα-mCit or mCit-PKCα in the absence of liposome (**Fig 4A; S1 Table**). The time-resolved anisotropy results were converted to steady-state anisotropy values by fluorescence lifetime-weighted averaging of the best-fit correlation times. Orthogonally, the actual steady-state anisotropy was measured for the corresponding conditions. We find good agreement between the two values and note that depolarization due to homoFRET results in a lower steady-state anisotropy value as expected **(Fig 4B)**. We use this finding to justify the use of steady-state anisotropy measurements to assess homoFRET in subsequent experiments. Next, we monitored a time course of steady-state anisotropy of C1a - 10 nm—C2 being sequentially treated with free calcium and EGTA. Within seconds of free calcium injection anisotropy decreases and resolves to near its starting point within seconds as EGTA is injected **(Fig 4C)**. This observation is consistent with an interpretation that C1a - 10 nm—C2 clusters to enable homoFRET in solution in the presence of free calcium and disassembles upon free calcium chelation.

### Self-assembly of C1a - 10 nm—C2 is rapid

**Fig 5:**
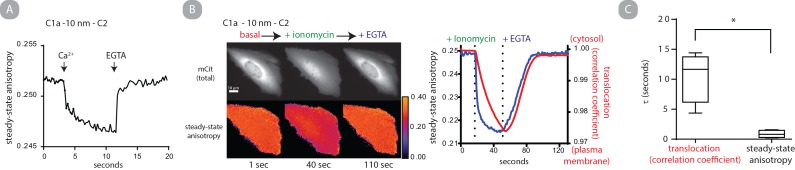
C1a - 10 nm—C2 self-assembles on a signaling relevant timescale. A.) C1a - 10 nm—C2 (200 nM) following addition of free calcium and EGTA. Steady-state mCit fluorescence anisotropy was measured using a polarized beam splitter at 5 fps. B.) Representative mCit total intensity and corresponding anisotropy heat map images of a CHO cell overexpressing C1a - 10 nm—C2 at three sequential time points after the addition of ionomycin and EGTA into the cellular media (left). The corresponding mean steady-state anisotropy value for each image in the time series (blue) and the Pearson’s correlation coefficient of each mCit total intensity image compared to the initial image in the time lapse (red), images obtained at 5 fps. B.) is representative of 3 independent experiments. C.) Cells overexpressing C1a - 10 nm—C2 were treated with ionomycin and monitored overtime (2 fps). Correlation coefficient and anisotropy were fit to a single exponential decay function and τ values for each pairwise measurement from 4 independent experiments are shown.

We next attempted to address the kinetics of self-assembly on the time scale of PKC signaling (milliseconds to seconds) [11]. We monitored the kinetics of C1a - 10 nm—C2 because homoFRET could be readily detected in the absence of liposomes. We used a polarized beam splitter coupled with an EMCCD camera to simultaneously obtain both polarization measurements at a rate of 5 fps. Even with the increased frame rate, kinetics of initial calcium stimulated assembly and disassembly exceeded the temporal resolution **(Fig 5A)**. Instead, we compared the relative rate of self-assembly compared to the rate of cellular translocation. CHO cells expressing C1a - 10 nm—C2 were sequentially perfused with the calcium ionophore ionomycin and cellular media containing excess EGTA while steady-state anisotropy was monitored. From the two polarized images obtained, a third image was generated, the mCit total, which is insensitive to changes in polarization [19]. The Pearson’s correlation coefficient between each mCit total image and the original mCit total image in a time course was calculated and used as a metric for subcellular translocation. When comparing the two metrics the rate of self-assembly preceeds translocation while disassembly and re-localization occur with similar rates **(Fig 5B)**. The pairwise obtained τ of self-assembly and translocation was 0.86 ± 0.63 s and 10.52 ± 4.03 s (mean and standard deviation; N = 4) respectively **(Fig 5C)**. This data indicates that self-assembly occurs on time scales consistent with PKC signaling in a cellular environment.

### PI(4,5)P_2_ clusters PKCα on liposomes independent of the C1a domain

**Fig 6:**
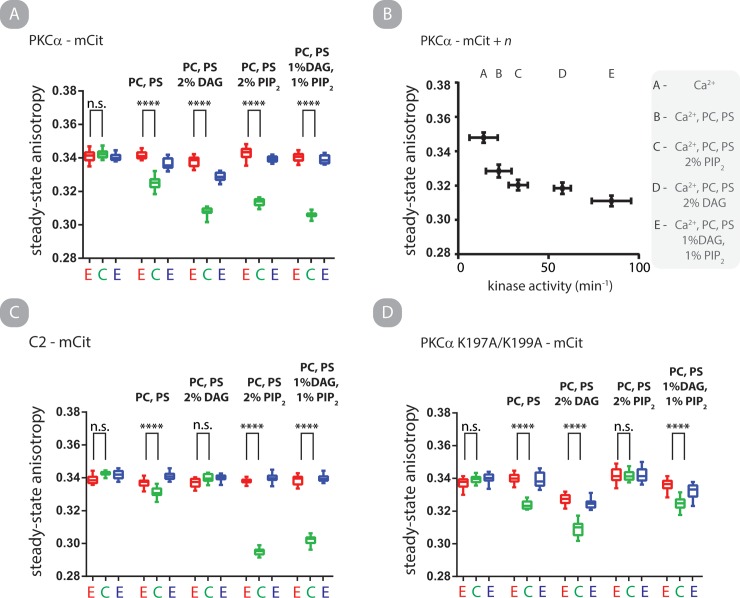
PKCα clusters on liposomes through two independent mechanisms, one driven by DAG and one by PI(4,5)P_2_. A—D) Steady-state anisotropy of PKCα-mCit (A, B) C2-mCit (C) and PKCα K197A/K199A-mCit (D) under the indicated conditions. All liposomes were matched with an 80:1 molar ratio of PS:PKCα where PS is 10% molar composition of liposome. Box and whisker represent min, max, mean, 25 and 75 percentiles of N ≥ 8 independent experiments. B.) Specific kinase activity of PKCα-mCit under otherwise matched conditions is plotted against steady-state anisotropy in the presence of free calcium. Shown is mean and SEM of N ≥ 6. All protein at 100 nM.

Finally, we assessed both catalytic activity and steady-state anisotropy of PKC-mCit-FLAG with different lipid compositions. We observe that liposomes containing either DAG or PI(4,5)P_2_ result in a calcium-induced decrease in fluorescence anisotropy. It is well established that *in vitro* catalytic activity of PKC is strongly dependent on the molar composition of liposomes [33]. Further, it has been suggested that *in vitro* catalytic activity of PKC may be sensitive to 2-dimensional organization of lipids on the surface of liposomes [33, 34]. We assessed homoFRET of PKC-mCit-FLAG in combination with liposomes with altered compositions (**Fig 6A**). The addition of calcium results in liposome composition dependent FRET, which is fully reversible upon chelation of free calcium (Fig 6A). The τ for assembly and disassembly are 12.9 ± 1.7 and 10.7 ± 0.5 seconds, respectively (mean and standard deviation; N = 7) **(S7 Fig)**. In parallel, kinase activity assays were performed with identical liposome compositions and PKC:liposome ratios (Fig 6B). We observe a correlation between homoFRET and specific kinase activity, suggesting clustering positively regulates kinase activity.

We were surprised that liposomes containing PI(4,5)P_2_ in the absence of DAG elicited high specific activity and homoFRET as DAG is typically considered a required co-factor for PKCα activity. However, several recent reports have demonstrated that PI(4,5)P_2_ (i) is a potent activator of PKCα *in vitro* [35], (ii) forms clusters in liposomes in the presence of free calcium [36] and, (iii) induces clustering of PS if calcium and PKCα C2 domain are present (which binds both PS and PI(4,5)P_2_) [23]. The latter two observations suggest that PI(4,5)P_2_ induce clustering of PKCα independently of a C1a-C2 interaction. We tested this by monitoring steady-state anisotropy of C2-mCit-FLAG under matched conditions (**Fig 6C**). HomoFRET for the C2 construct is observed if PI(4,5)P_2_ is present in the liposome. We next inserted point mutations to specifically disrupt the C2—PI(4,5)P_2_ interaction [13]. HomoFRET of PKC-mCit-FLAG K197A/K199A is abrogated in solution with liposomes containing PI(4,5)P_2_ while not affecting the response from liposomes containing DAG (**Fig 6D**). These results provide positive evidence that clustering of PKC on liposomes *in vitro* is dependent on calcium as well as DAG or PI(4,5)P_2_ binding.

## Conclusions

We here report the *in vitro* phenomenon that PKCα self-assembles specifically, rapidly and reversibly in the presence of calcium. We find that the C1a and C2 domains are required for self-assembly. These domains have been previously reported to interact but under contradictory conditions [29, 30]. In the study by the Cho group, it was anticipated that an intramolecular interaction between the two domains is present in the absence of calcium, but that the interaction is abrogated upon lipid binding [29]. In contrast, in a study by the Stubbs group, a high affinity intermolecular interaction (4 nM) was reported between C1a and C2 domains in the presence of calcium and phorbol ester [30]. Our present findings support both an intramolecular and intermolecular interaction between C1a and C2 domains (Figs 3 and 4). Our intramolecular FRET data directly support an intramolecular interaction between the C1a and C2 domains in the absence of calcium (Fig 3D). Upon calcium binding, intermolecular self-assembly of C1a and C2 occurs (Figs 3–5). The degree of self-assembly of C1a-C2 can be tuned if the intramolecular C1a-C2 interaction is partially or fully disrupted (Fig 3). We speculate that distinct cellular locations, including local plasma membrane environments may tune the intramolecular C1a-C2 complex, and in that way self-assembly may be regulated. To probe this possibility, we explored the degree by which self-assembly of full-length PKC is regulated by lipid composition on liposomes *in vitro*. We found that the degree of self-assembly of PKC-mCit measured by homoFRET can be regulated by both DAG and PI(4,5)P_2_ (Fig 6). We found that a higher degree of self-assembly on liposomes generally correlates with higher kinase activity (Fig 6B). These data support the potential role of a regulatory intramolecular interaction between the C1a and C2 domain in tuning PKC function.

**Fig 7:**
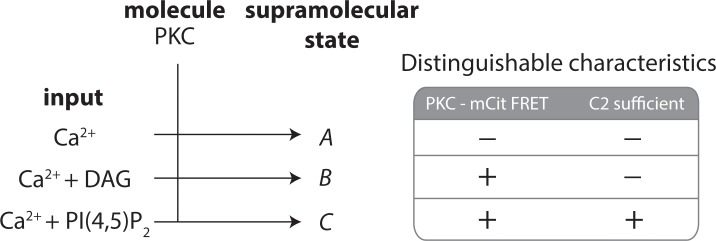
PKC forms distinguishable supramolecular states with different combinations of inputs. Using two experimental criteria PKC can be parsed into three unique supramolecular states. FRET puts a physical constraint on the proximity of PKC molecules within ~1.6 of *R*_*o*_ (mCer-mCit *R*_*o*_ = 5.4 nm; 8.6 nm) suggestive of direct interactions [38], but is sensitive to the orientation between chromophore dipoles such that the absence of FRET does not rule out an interaction. Calcium alone causes self-assembly of PKC which can be directly observed using biophysical approaches (Fig 1), is not detectable by FRET (Fig 4), and requires both the C2 and C1a domain (Figs 2 and 3). Calcium plus liposomes containing DAG result in intermolecular FRET between PKC–mCit (Fig 4), but require the full-length protein and not just the C2 domain (presumably requires C1 domains which are only known DAG binding sites; Fig 6). Calcium plus liposomes containing PI(4,5)P_2_ result in intermolecular FRET and the C2 domain is sufficient for this result (Fig 6). Schematic is organized to parallel schematics initially describing supramolecular structures proposed by John-Marie Lehn [39].

It remains unknown (i) if the calcium stimulated self-assembly of PKC phenomenon occurs in cells and (ii) what biological functions it may be involved in. Previously, we provided evidence supporting the functional relevance of homo-oligomerization of PKCα in LPA and PMA stimulated phosphorylation of ERK 1/2 in CHO cells [14]. Here we provide homoFRET data indicating that the C1a and C2 domains can self-assemble in cells (Fig 5). While FRET-based methodologies can be useful in assessing if an interaction occurs, it can be challenging to assess other characteristics including stoichiometry and distribution of oligomeric states without complimentary data. In this report we have deliberately chosen to study homo-oligomerization of PKCα in the absence of lipids so that methodologies based on hydrodynamic properties of PKC (DLS, size exclusion chromatography, differential sedimentation) or spatially resolved fluorescence intensity can provide insight into this phenomenon and complement studies of PKC in the presence of lipids/liposomes. What can be clearly distinguished in our current report is that PKC is capable of clustering in a calcium specific manner independent of lipid components (Fig 1). Despite this, lipid components, namely DAG and PI(4,5)P_2_, play critical roles in the self-assembly of PKC in the presence of calcium (Fig 6). We speculate that clustering may be a mechanism that modulates spatial-temporal regulation of PKC in select physiological situations. It should be noted that calcium stimulated self-assembly is typically not fully reversible on the time scales we have investigated, although it is not yet clear if this will have physiological consequences. We are intrigued by the shared characteristics between self-assembly of PKCα and ideal information processing molecules, as defined in the field of supramolecular chemistry [37]. In line with this, our data distinguish three unique supramolecular states of PKC formed upon calcium binding which may each have unique functional properties (**Fig 7**).

We speculate that PKCα self-assembly may be critical in the spatial organization of dynamic signaling hubs. It has been previously demonstrated that the PKCα C2 domain clusters into diffraction limited punctae on the plasma membrane, a phenomenon dependent on PIP_2_ binding [13]. Corresponding *in vitro* reports suggest that PIP_2_ clusters on the surface of liposomes in the presence of calcium [36] and the PKC C2 domain can additionally facilitate clustering of PS [23]. Our current finding that PIP_2_ plus calcium leads to PKC clustering (Fig 6) is consistent with a model in which PIP_2_ clusters can focally accumulate PKC and molecules which directly bind to PKC (PS, DAG, substrate et. ct.). A recent report demonstrated how PKC activation and phosphorylation of MARCKS protein, which sequesters PIP_2_ unless phosphorylated by PKC, can lead to accumulation and activation of PI3K to newly exposed PIP_2_ on supported lipid bilayers *in vitro* [40]. Such a finding in conjunction with clustering may provide a mechanism by which PKC can generate and spatially organize a signaling hub with a mechanism independent of direct scaffolding. Numerous other signaling and cytoskeletal proteins including calcium insensitive PKC, AKT and vinculin also interact with PIP_2_, however on time scales anticipated to be slower than calcium sensitive PKCs [41]. The ability for PKC to self-assemble on the surface of the plasma membrane and locally phosphorylate substrate (e.g. MARCKS) may facilitate localized recruitment of signaling molecules. A recurring phenomenon in PKC biology, especially in calcium sensitive PKCs, is the role in spatially organizing membrane localized signaling hubs at, for example, focal adhesion complexes (both focal adhesion kinase and vinculin bind to and cluster with PIP_2_ *in vitro* [42, 43]), or cell-cell junctions [10]. We speculate that self-assembly of PKC can be functional interdependently of kinase activity by regulating the spatial localization of other signaling molecules on short or long time scales.

## Supporting Information

S1 Fig D246N point mutation blocks calcium induced oligomerization of PKCα-mCit. Size exclusion chromatography elution profile of PKCα-mCit wild-type (A) or D246N (B) under indicated incubation conditions. (C) Differential fractionation of the same fusion proteins following a 30 min incubation with or without 300 μM free calcium. The soluble (S) and pelleted (P) fractions were run on an SDS-PAGE gel and imaged on a typhoon imager for mCit fluorescence. The fluorescent degradation products observed in gel-filtration will have effects on the quantification of some of the other experimental results. As protein concentration is normalized by mCitrine fluorescence, the concentration of intact PKC protein will be less than anticipated. (i) For DLS, degradation components will lead to a lower ensemble mass predicted in both the EGTA and Calcium conditions. (ii)_For time resolved fluorescence anisotropy it will lead to the ‘fraction 1’ parameter being artificially lower by the degree of fluorophores that are incapable of self-assembling. (iii) For the coverslip assay, it will decrease the intensity of PKC punctae Overall, it will lead to an underestimation of the effects of self-assembly.
(EPS)

S2 Fig Representative analysis of DLS data of PKCα-mCit in EGTA buffer. (A) The intensity autocorrelation data (blue dots) is fit by a regularization model (black line) with residuals in bottom panel. (B) An isotropic spheres model is applied to calculate the distribution of particle mass. This panel is representative of most experiments where two populations are observed, the predominant peak near the expected mass of particles, and a second peak of much larger particles that was not removed despite high-speed ultracentrifugation immediately prior to the measurement.
(EPS)

S3 Fig Two step growth of PKC oligomers. (A) The number of identified spots on a coverslip of PKCα-mCit (100 nM) with and without free calcium was assessed at time points up to 60 minutes. The data are fit to a linear regression for visualization. The points represent mean and standard deviation of 4 images (left). A cumulative histogram of the mean intensity of each spot normalized to the total intensity of the image from the corresponding images (right). (B) The concentration of PKCα-mCit was held constant (50 nM) and unlabeled PKCα (0, 50, 100, 200, 400 nM) was added such that the concentration of PKCα (represented on the x-axis) is the sum of the two species. The mean spot number and the mean intensity with standard deviation (6 images) of all the spots are plotted at 3 time points after the addition of free calcium. The mean intensity of the 450 nM PKCα condition at the 60 and 180 minute time points are not reported due to saturation of spots in the imaging conditions. (C) A schematic depicting the two processes in PKC self-assembly observed. The initial increase in spot number is the result of nucleation events. The subsequent decrease in spot number as well as the increase in mean particles size is the result of smaller oligomers joining into larger oligomers.
(EPS)

S4 Fig Depletion of lipids in clarified HEK lysate. This experiment was conducted alongside the representative gel images in Fig 1E. A constant concentration of DiI, a lipophilic membrane stain, was added to serial dilutions of a 1 mg/mL stock solution of POPC sonicated in lysis buffer. The sample was excited at 521 nm and DiI emission was monitored from 550 nm– 610 nm. A matched DiI free spectra was measured and subtracted from the DiI sample.The background corrected spectra was integrated and plotted above. The serial dilution is fit to a linear curve (black line). A matched protocol was applied to the HEK lysate and clarified HEK lysate. The measured DiI intensities are represented by the dashed lines.
(EPS)

S5 Fig Separating the C1 and C2 domains eliminates calcium clustering on coverslip. (A) A schematic shows the modular strategy by which we previously created three PKCα biosensors. All three biosensors specifically clustered in the presence of calcium. (B) We then used TEV protease to site specifically generate two peptide fragments, the N-terminus always containing the mCer, and the C-terminus the mCit fluorophore. We then took two images of each sample using a dual view emission splitter optimized for mCer and mCit, with two excitations optimized for mCer and mCit. In total 4 images of each field of view were obtained (mCer excitation: mCer emission; mCer excitation: mCit emission; mCit excitation: mCer emission; mCit excitation: mCit emission) of which we only analyzed the mCer excitation: mCer emission, and mCit excitation: mCit emission images which had negligible cross excitation and bleed through for the purposes of our spot identifier program. The spot numbers for both channels are depicted normalized to the mean number of spots in the corresponding non-TEV treated sample (4 images in each channel/ condition).
(EPS)

S6 Fig D55A and R252A do not disrupt C1a –C2 interactions. Experiment were performed as described in Fig 3E with the indicated point mutants.
(EPS)

S7 Fig Reversible FRET between full-length PKC on liposomes. Bi-molecular heteroFRET between the indicated protein (40 nM donor and 120 nM acceptor) was monitored following the sequential addition of buffer containing free calcium (green) or EGTA (black). Liposomes were made from porcine polar brain extract with 2% w/w DAG as described previously [14]. Shown is the mean and standard deviation of N = 7 experiments. Association and dissociation were fit to single exponential functions (not shown). The τ’s for assembly and disassembly is 12.9 ± 1.7 and 10.7 ± 0.5 seconds, respectively (mean and standard deviation).
(EPS)

S1 Table Values from TCSPC and DWR analysis. Fluorescence lifetime of the sample obtained at the magic angle was fit by a single exponential function. The lifetime value was constrained as the anisotropy profile was fit to a 2-exponential function where the two correlation time parameters and the fractional contribution of the faster correlation time were co-estimated. Initial and final anisotropies were constrained to 0.4 and 0, respectively. For the conditions with only one reported correlation time, only a single correlation time was resolved by the fit (i.e., the fraction of the faster component was zero). Steady-state anisotropy was calculated as the fluorescence weighted average of the anisotropy decay given by the best-fit parameters. Standard errors of the fit (67% confidence interval) are listed in brackets.
(DOCX)

S2 Table Data used for statistics throughout the manuscript. (XLSX)

S1 Text Analysis supplemental to results in Fig 1. (DOCX)
