# Supplementary material for: Calcium Stimulates Self-Assembly of Protein Kinase C α In Vitro
Source: PLoS One. 2016 Oct 5;11(10):e0162331. doi: 10.1371/journal.pone.0162331 (PMC5051681; doi:10.1371/journal.pone.0162331)
Supplement: S1 Table — Fluorescence lifetime of the sample obtained at the magic angle was fit by a single exponential function. The lifetime value was constrained as the anisotropy profile was fit to a 2-exponential function where the two correlation time parameters and the fractional contribution of the faster correlation time were co-estimated. Initial and final anisotropies were constrained to 0.4 and 0, respectively. For the conditions with only one reported correlation time, only a single correlation time was resolved by the fit (i.e., the fraction of the faster component was zero). Steady-state anisotropy was calculated as the fluorescence weighted average of the anisotropy decay given by the best-fit parameters. Standard errors of the fit (67% confidence interval) are listed in brackets. (DOCX) [file pone.0162331.s008.docx]

Supplemental Table:

|  | Fluorescence lifetime (ns)  $\tau$ | Correlation time 1 (ns)  $\phi_{1}$ | Fraction correlation time 1 $\chi_{1}$ | Correlation time 2  (ns)  $\phi_{2}$ |
| --- | --- | --- | --- | --- |
| mCit-PKCα (EGTA) TCSPC | 3.43  [3.42 to 3.45] | -- | -- | 32.6  [30.0 to 35.9] |
| mCit-PKCα (Ca^2+^) TCSPC | 3.43  [3.41 to 3.45] | -- | -- | 36.0  [32.6 to 40.1] |
| PKCα-mCit (EGTA) TCSPC | 3.44  [3.42 to 3.46] | 0.753  [0.343 to 1.529] | 0.070  [0.048 to 0.114] | 30.5  [25.9 to 41.4] |
| PKCα-mCit (Ca^2+^) TCSPC | 3.45  [3.42 to 3.46] | 0.753  [0.0 to 0.93] | 0.065  [0.055 to 0.076] | 32.1  [29.0 to 35.9] |
| PKCα-mCit + liposome (EGTA) TCSPC | 3.46  [3.44 to 3.48] | -- | -- | 31.2  [28.5 to 36.0] |
| PKCα-mCit + liposome (Ca^2+^) TCSPC | 3.28  [3.26 to 3.29] | 0.761  [0.372 to 1.34] | 0.182  [0.127 to 0.283] | 12.1  [9.84 to 17.4] |
| PKCα-mCit (EGTA) DWR | 3.41  [3.39 to 3.43] | 0.431  [0.0 to 0.692] | 0.088  [0.065 to 0.097] | 40.8  [31.8 to 50.7] |
| PKCα-mCit (Ca^2+^) DWR | 3.41  [3.39 to 3.43] | 0.349  [0.0 to 0.577] | 0.093  [0.071 to 0.102] | 41.5  [32.1 to 51.1] |
| PKCα-mCit + liposome(EGTA) DWR | 3.42  [3.40 to 3.44] | 0.540  [0.217 to 0.760] | 0.094  [0.0711 to 0.109] | 43.5  [34.0 to 53.6] |
| PKCα-mCit + liposome (EGTA + Ca^2+^) DWR | 3.30  [3.28 to 3.32] | 0.554  [0.428 to 0.685] | 0.230  [0.208 to 0.254] | 30.9  [25.5 to 40.4] |
| PKCα-mCit + liposome (EGTA + Ca^2+^ + EGTA) DWR | 3.34  [3.32 to 3.36] | 0.525  [0.342 to 0.746] | 0.152  [0.131 to 0.177] | 40.8  [32.6 to 52.2] |
| C1a-SPASM-C2 (EGTA) DWR | 3.30  [3.28 to 3.33] | 0.356  [0.178 to 0.563] | 0.150  [0.130 to 0.175] | 61.2  [43.4 to 110] |
| C1a-SPASM-C2 (Ca^2+^) DWR | 3.18  [3.15 to 3.20] | 0.225  [0.063 to 0.349] | 0.236  [0.208 to 0.259] | 129.7  [62.2 to >1000] |

STable 1: **Values from TCSPC and DWR analysis.** Fluorescence lifetime of the sample obtained at the magic angle was fit by a single exponential function. The lifetime value was constrained as the anisotropy profile was fit to a 2 exponential function where the two correlation time parameters and the fractional contribution of the faster correlation time were co-estimated. Initial and final anisotropies were constrained to 0.4 and 0 respectively. For the conditions with only one reported correlation time, only a single correlation time was resolved by the fit (i.e., the fraction of the faster component was zero). Steady-state anisotropy was calculated as the fluorescence weighted average of the anisotropy decay given by the best-fit parameters. Standard errors of the fit (67% confidence interval) are listed in brackets.
