# Supplementary material for: Calcium Stimulates Self-Assembly of Protein Kinase C α In Vitro
Source: PLoS One. 2016 Oct 5;11(10):e0162331. doi: 10.1371/journal.pone.0162331 (PMC5051681; doi:10.1371/journal.pone.0162331)
Supplement: S1 Text — (DOCX) [file pone.0162331.s010.docx]

Calcium stimulates self-assembly of Protein Kinase C α *in vitro*

Carter J. Swanson, Ruth F. Sommese, Karl J. Petersen, Michael Ritt, Joshua Karslake, David D. Thomas, Sivaraj Sivaramakrishnan

**Supplemental results:**

*Size exclusion chromatography*

We started by assessing PKCα oligomerization by SEC. PKCα-mCit under EGTA buffered conditions ran as 1 prominent peak followed by several smaller degradation product peaks **(SFig. 1a)**. The MW of the prominent peak was determined to be 1.00*10^5^ ± 0.19*10^5^ dalton (mean and 1 standard deviation from Gaussian fit; anticipated MW of PKCα-mCit is 1.06*10^5^ dalton), and PKC-α was detected in the corresponding fraction by a monoclonal anti-PKCα antibody. When the mobile phase contained 300 µM free Ca^2+^ and the protein sample was pre-incubated with matched buffer for 3, 10 or 30 minutes prior to the chromatography run, the prominent peak was progressively quenched and no additional peak was observed. The column manufacturer states the exclusion limit of the resin (Superdex 200; for globular protein) as 1.3 * 10^6^ daltons (~10 fold increase in Mr of monomer assuming spherical particles). Interestingly, no oligomers within the range of 2 – 10 times the monomer size were resolved, except for a small peak in the void fraction after the shortest calcium incubation period. We conclude that in the presence of calcium the protein oligomerizes into particles larger than the exclusion limit of the column. We were surprised that oligomeric species could not be resolved in the range of 1.14*10^5^ - 1.3 * 10^6^ daltons (2 - 10 molecules/particle). To address specificity, we introduced the single point mutation D246N which is known to disrupt Ca^2+^ ion coordination to the C2 domain([1](#_ENREF_1)). We find that under EGTA buffered conditions the D246N point mutation has no observable effect on Mr compared to the native sequence **(SFig. 1b)**. However, when treated with Ca^2+^, D246N runs exactly as in the EGTA buffered condition. This finding provides evidence that not only is Ca^2+^ minimally sufficient to oligomerize PKCα, it does so specifically through the well-characterized Ca^2+^ binding pocket in the C2 domain.

*Differential sedimentation*

We chose centrifugation conditions such that the monomer (MW < 112.6 KDa) species would remain in the supernatant, while any aggregated/oligomeric species would be pelleted. We found that protein buffered with EGTA primarily remained in solution, while buffering with 200 µM free Ca^2+^ caused a significant pellet fraction to be observed. However, if the D246N mutation is present, the protein no longer forms pellets in a calcium-dependent manner (**S Fig. 1c**). We designed an experiment in which the same protein sample was sequentially buffered with EGTA, fractionated, the soluble component buffered with free Ca^2+^, re-fractionated, the pellet re-suspended in EGTA buffered solution and fractionated a 3^rd^ time. Remarkably, we observed calcium to drive pelleting of the protein, but once the pellet was re-suspended in EGTA buffered solution the entire fraction remained in the soluble fraction **(Fig. 1a)**. This experimental result suggests that oligomerization is reversible and dependent on calcium.

*Dynamic Light Scattering*

In order to get an estimate of the size of oligomers as well as confirm reversibility, we utilized dynamic light scattering (DLS). We initially investigated a modestly concentrated sample of PKCα-mCit (1 µM) buffered with EGTA so that the hydrodynamic radius between SEC and DLS experiments (H_R(SEC)_, H_R(DLS)_) could be compared. Using a regularization fit, it was found that PKCα-mCit was bi-model, with a peak with estimated H_R_ consistent with monomers (in all cases representing > 94% mass) as well as a peak with H_R_ ~100 fold larger than monomer (presumably aggregated protein) **(SFigure 2)**. Not surprisingly, the monomeric peak is polydisperse, which is consistent with SEC data in which several degradation products were observed. When the H_R(DLS)_ of the first peak (3.14 nm) is used to estimate a molar mass using the Stokes-Einstein relationship with an isotropic spheres model, the mean is 95.8 ± 16.3 KDa (mean and s.e.m. n = 65; compared with 100 KDa from SEC and 106 KDa estimate from primary sequence). We then sequentially added calcium and EGTA to the same sample with 15 min incubations in between. A dramatic shift in the autocorrelation data towards longer time occurred following calcium addition and partial recovery to shorter time with EGTA addition **(Fig. 1 b left)**. We used a cumulant fit to estimate an averaged diffusion constant and used an isotropic sphere model to calculate the MW of all species in the sample and normalized by the starting condition. The average mass sequentially increased by 13 fold and returned to 4 fold above the starting EGTA buffered condition **(Fig. 1 b right)**. The introduction of the D246N point mutation increased a modest 1.7 fold following introduction of calcium. These results are consistent with both the specificity and reversibility of the oligomerization phenomenon previously observed.

*Fluorescence microscopy*

Using non-specific adhesion to coverslips, PKCα-mCit formed large fluorescent punctae specifically and reversibly in the presence of free calcium **(Fig. 1 c)**. By quantifying the fluorescent images the relative number and intensity of particles could be distinguished. We initially aimed to do step-wise photobleaching of the punctae to estimate the # of molecules / punctae, however the intensity of punctae >> the intensity of individual fluorophores making this quantification unfeasible as punctae are saturated under imaging conditions resolving individual fluorophores. Instead, we quantified the total fraction of molecules in punctae **(Fig. 1 c right)** as well as the heterogeneity in punctae intensity. Further, these punctae are used to justify the isotropic models used in SEC and DLS analysis as they visually appear as isotropic spots as opposed to fibers. As a control, the occurrence of punctae as well as differential sedimentation was quantified as a function of free Ca^2+^ concentration **(Fig. 1d)**. Both punctae formation and the degree of sedimentation were variable with the free calcium concentration and could be fit to a one phase binding curve (*K_D_* = 1.6 ± 0.3 µM punctae formation, *K_D_* = 320 ± 260 nM differential fractionation). Both fall in the range of reported C2·Ca^2+^ equilibrium coefficients ([1-3](#_ENREF_1)). This assay was further used to explore additional axis of this phenomenon including time and protein concentration. We observed that both punctae number and the relative intensity of punctae increases as a function of time, and that higher PKCα concentration accelerates the growth of punctae **(SFig. 3)**. These results suggest a mode of growth in which two processes occur: nucleation of an oligomer from monomers as well as the joining of oligomers into larger oligomers. Such a process is more commonly referred to as polymerization or self-assembly than oligomerization. Because the polymerization is strictly dependent on calcium we will refer to this phenomenon as self-assembly. Together these data support our conclusion that self-assembly of PKCα is reversible and specific to calcium binding.

**References:**

1. Medkova, M., and Cho, W. (1998) Mutagenesis of the C2 domain of protein kinase C-alpha. Differential roles of Ca2+ ligands and membrane binding residues. *J Biol Chem* **273**, 17544-17552

2. Kohout, S. C., Corbalan-Garcia, S., Torrecillas, A., Gomez-Fernandez, J. C., and Falke, J. J. (2002) C2 domains of protein kinase C isoforms alpha, beta, and gamma: activation parameters and calcium stoichiometries of the membrane-bound state. *Biochemistry* **41**, 11411-11424

3. Sanchez-Bautista, S., Marin-Vicente, C., Gomez-Fernandez, J. C., and Corbalan-Garcia, S. (2006) The C2 domain of PKCalpha is a Ca2+ -dependent PtdIns(4,5)P2 sensing domain: a new insight into an old pathway. *J Mol Biol* **362**, 901-914
